# SoSyM reflections: the 2020 "State of the Journal" report

**DOI:** 10.1007/s10270-021-00871-4

**Published:** 2021-02-17

**Authors:** Huseyin Ergin, Jeff Gray, Bernhard Rumpe, Martin Schindler

**Affiliations:** 1 Ball State University, Muncie, USA; 2 University of Alabama, Tuscaloosa, AL USA; 3 RWTH Aachen University, Aachen, Germany

When writing the “2019 State of the Journal Report” at this same time last year, we could not have predicted the global changes that would beset us with so many challenges from the emergence of the COVID-19 pandemic. Some of us lost loved ones, friends, and colleagues, while the entire world adapted to working from home, participating in remote classrooms, and adopting safety precautions as a new means of daily life. The research community was also affected, with the cessation of travel leading to virtual conferences. Thanks to the heroic efforts of many conference chairs^1^ adapting to the quick pace of change, scientific discussions continued, but sometimes in a less personable form. Journals also experienced changes with submissions on the rise, but fewer reviewers available to assist with the evaluation because of personal challenges faced by many.

Yet, in the presence of a global pandemic, scientific contributions continued in the software and systems modeling community. The number of SoSyM submissions over the past year saw an increase, while the general health of the journal remains strong. Measures put in place recently, such as the second year of moving to six issues per year, have helped to reduce the time to publication significantly. The Open Access movement is also progressing, with Springer announcing new initiatives to make SoSyM publications more accessible to a broader community of researchers. The rest of this editorial summarizes the progress made by the journal during this unprecedented situation.

Our hope is that you remain safe and have a productive 2021!

## 2020 summary statistics

The six SoSyM issues published in 2020 contained 17 Regular papers, 33 Special Section papers, 6 Theme Section papers, 4 Expert Voices, and 9 Guest Editorials. In total, 1587 pages were published in volume 19.

We are very happy to report that the 2-year Impact Factor (IF) for SoSyM continues to be very respectable at 1.876 (previously at 2.66 in 2019 and 1.722 in 2018), with the 5-year IF holding at 1.915. Furthermore, the h-5 Google Scholar ranking places SoSyM at #12 among all conferences and journals related to software engineering and programming languages.

There are two new positive “records” set for SoSyM in 2020 related to submissions and downloads. Over the past year, SoSyM received 372 submissions—the largest number for any year in our history (over 100 submissions over the average of the past three years). Furthermore, at the time of this writing, there were over 164,316 downloads from January through the end of November 2020. This is well beyond the 2019 download numbers (which was also a record at that time) of 136,378. These two observations suggest the growing interest in SoSyM among the research community.

The acceptance rate in 2020 was 19%, which is a respectable rate for a high-quality journal such as SoSyM. The average time from submission to the final decision (accept or reject) has slightly increased to 146 days (128 days in 2019 and 138 days in 2018).

## SoSyM's ten-year most influential paper Awards

MODELS 2020 was originally planned to be held in Montreal, Canada, but due to the safety concerns with COVID-19, the conference was held virtually in October 2020. We thank the MODELS 2020 organizers (Eugene Syriani and Houari Sahraoui) and the PC co-Chairs (Silvia Abrahão and Juan de Lara) for collaborating with SoSyM. We were able to host another awards session with paper presentations of the Best SoSyM Papers over the past 10 years during a special virtual session of MODELS 2020. The selection was based on the ISI citation index among papers published in SoSyM since 2010. More information about the awards can be found at: http://www.sosym.org/awards/.

The **SoSyM 2020 "Ten-year most influential Regular paper award"** was given to:

- Jeff Offutt and Ye Wu, "Modeling presentation layers of web applications for testing," In: *Journal on Software and Systems Modeling (SoSyM)*, Volume 9, Issue 2, pp. 257–280, Springer, April 2010.

- https://doi.org/10.1007/s10270-009-0125-4

The **SoSyM 2020 "Ten-year most influential Theme Section paper award"** was given to:

- Nicolas Anquetil, Uira Kulesza, Ralf Mitschke, Ana Moreira, Jean-Claude Royer, Andreas Rummler, and Andre Sousa, "A model-driven traceability framework for software product lines," In: *Journal on Software and Systems Modeling (SoSyM)*, Volume 9, Issue 4, pp. 427–451, Springer, September 2010.

- https://doi.org/10.1007/s10270-009-0120-9

### SoSyM's Journal-First papers at MODELS 2020

The continuous collaboration between SoSyM and the MODELS conference in organizing the SoSyM “Journal-First” was successfully continued. This enables authors of recent SoSyM papers to present their work across the core conference sessions at MODELS. Through this collaboration, SoSyM authors have the opportunity to reach a broader audience to present their work. This also benefits the MODELS conference program by including research talks that explore more depth through analytical and empirical evidence than can be presented in a traditional conference submission. The virtual nature of MODELS 2020 allowed for more SoSyM papers to be selected for presentation. At MODELS 2018, four articles were presented. At MODELS 2019, we increased this number to seven articles and at MODELS 2020 to ten articles (papers that were accepted from July 2019 through June 2020). The SoSyM “Journal-First” papers presented at MODELS 2020 were the following (note: Some of the papers are available online but have not yet received an assignment to an issue):

- Yinling Liu, Tao Wang, Haiqing Zhang, and Vincent Cheutet. “An improved approach on the model checking for an agent-based simulation system.” In: *Journal on Software and Systems Modeling (SoSyM)*, Springer, in press, 2020. https://doi.org/10.1007/s10270-020-00807-4
- Simin Cai, Barbara Gallina, Dag Nyström, and Cristina Seceleanu. “Specification and automated verification of atomic concurrent real-time transactions.” In: *Journal on Software and Systems Modeling (SoSyM)*, Springer, in press, 2020. https://doi.org/10.1007/s10270-020-00819-0
- Karim Jahed, Mojtaba Bagherzadeh, and Juergen Dingel. “On the benefits of file-level modularity for EMF models.” In: *Journal on Software and Systems Modeling (SoSyM)*, Volume 20, Issue 1, Springer, January 2021. https://doi.org/10.1007/s10270-020-00804-7
- Stefan Götz, Matthias Tichy, and Raffaela Groner. “Claimed Advantages and Disadvantages of (dedicated) Model Transformation languages: A Systematic Literature Review.” In: *Journal on Software and Systems Modeling (SoSyM)*, Springer, in press, 2020. https://doi.org/10.1007/s10270-020-00815-4
- Anthony Anjorin, Thomas Buchmann, Bernhard Westfechtel, Zinovy Diskin, Zinovy, Hsiang-Shang Ko, Romina Eramo, Georg Hinkel, Leila Samimi-Dehkordi, and Albert Zuendorf. “Benchmarking bidirectional transformations: theory, implementation, application, and assessment.” In: *Journal on Software and Systems Modeling (SoSyM)*, Volume 19, Issue 3, pp. 647–691, Springer, May 2020. https://doi.org/10.1007/s10270-019-00752-x
- Milena Guessi, Flavio Oquendo, and Elisa Yumi Nakagawa. “Ark: A constraint-based method for architectural synthesis of smart systems.” In: *Journal on Software and Systems Modeling (SoSyM)*, Volume 19, Issue 3, pp. 741–762, Springer, May 2020. https://doi.org/10.1007/s10270-019-00764-7
- Enyo Gonçalves, Camilo Almendra, Miguel Goulão, João Araújo, and Jaelson Castro. “Using empirical studies to mitigate symbol overload in iStar extensions.” In: *Journal on Software and Systems Modeling (SoSyM)*, Volume 19, Issue 3, pp. 763–784, Springer, May 2020. https://doi.org/10.1007/s10270-019-00770-9
- Nicolas Hili, Mojtaba Bagherzadeh, Karim Jahed, and Juergen Dingel. “A model-based architecture for interactive run-time monitoring.” In: *Journal on Software and Systems Modeling (SoSyM)*, Volume 19, Issue 4, pp. 959–981, Springer, July 2020. https://doi.org/10.1007/s10270-020-00780-y
- Juan C. Vidal, Paulo Carreira, Vasco Amaral, Joao Aguiam, and João Sousa. “Towards high-level fuzzy control specifications for building automation systems.” In: *Journal on Software and Systems Modeling (SoSyM)*, Volume 19, Issue 3, pp. 625–646, Springer, May 2020. https://doi.org/10.1007/s10270-019-00755-8
- Bence Graics, Vince Molnár, András Vörös, István Majzik, and Dániel Varró. “Mixed-semantics composition of statecharts for the component-based design of reactive systems.” In: *Journal on Software and Systems Modeling (SoSyM)*, Volume 19, Issue 6, pp. 1483–1587, Springer, November2020. https://doi.org/10.1007/s10270-020-00806-5

## Changes to the editorial board

Each year, Editors who served the SoSyM community for many years retire from the SoSyM Editorship with distinction of service and our deep appreciation. In 2020, Dorina Petriu announced that she desired to step down as an Editor after many years of service. Thanks Dorina for all of your contributions to SoSyM! 
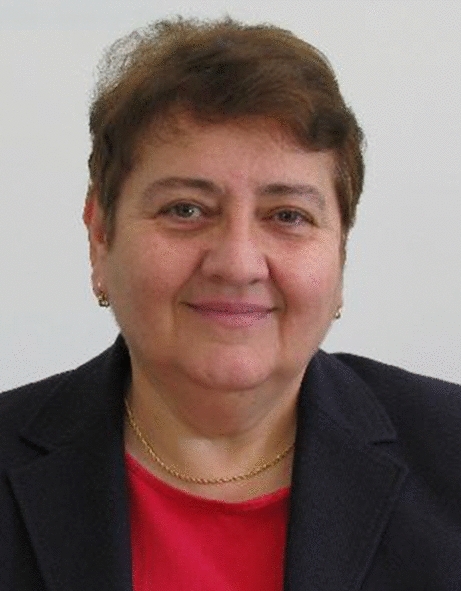


As mentioned earlier in this report, SoSyM received a record number of submissions this year (372), which requires us to grow the Editorial Board. We are very happy to welcome the following new SoSyM Editors and look forward to working with them in the future.

**Table:** 

				
Loli Burgueño	Jan Ringert	Andreas Wortmann	Tao Yue	Steffen Zschaler

## Reviewers in 2020

A strong research community depends on the efforts of volunteers who help serve as reviewers. The software and systems modeling community has always risen to the request for help from SoSyM. We appreciate all of the help that the reviewers provided in service to the modeling community! We would also like to offer special recognition to the following reviewers, who were recommended as the SoSyM Best Reviewers of 2020, based on the technical depth and feedback provided to authors over the past year—congratulations! We will send a certificate of recognition to each of the following reviewers:

- Juergen Dingel, Antonio García-Domínguez (twice!), Marcus Gerhold, Mario Gleirscher, Sylvain Hallé, Stefan Klikovits, Thomas Kühne, Hugo A. López, Florian Matthes, Libero Nigro, Bentley Oakes, Stefan Sauer, Harald Störrle, Valentín Valero, Steven van Kervel, and Simon Van Mierlo.

Below is a list of those who reviewed one or more papers for the journal in the last year. The complete list of reviewers can also be found on our website http://www.sosym.org/people/.

- Rasmus Adler, Paulo Alencar, Ian Alexander, Joao Paulo Almeida, Ahmad Salim Al-Sibahi, Juliana Alves Pereira, Vasco Amaral, Elske Ammenwerth, Moussa Amrani, Daniel Amyot, Kelly Androutsopoulos, Anthony Anjorin, Muhammad Anwar, Vincent Aranega, Joao Araujo, Paolo Arcaini, Chetan Arora, Cyrille Artho, Wesley K. G. Assuncao, Colin Atkinson, Vanessa Ayala-Rivera, Thomas Baar, Onder Babur, Mojtaba Bagherzadeh, Mira Balaban, Torsten Bandyszak, Luciano Baresi, Kamel Barkaoui, Jiri Barnat, Angela Barriga, Francesco Basciani, María Bastarrica, Dinesh Batra, Steffen Becker, Mitra Tabaei Befrouei, David Benavides, Luca Berardinelli, Gabor Bergmann, Simona Bernardi, Ilia Bider, Robert Bill, Olivier Biot, Karsten Boehm, Francis Bordeleau, Dominik Bork, Artur Boronat, Saida Boukhedouma, Frederic Boulanger, Erwan Bousse, Drazen Brdjanin, Uwe Breitenbuecher, Ruth Breu, Jean-Michel Bruel, Davide Brugali, Hugo Bruneliere, Andrea Burattin, Erik Burger, Loli Burgueño, Arvid Butting, Cristina Cabanillas, Josè Campos, Laurent Capocchi, Jan Carlson, Victorio Carvalho, Roberto Casadei, Ana Cavalcanti, Graziana Cavone, Moharram Challenger, Mohammad Chami, Michel Chaudron, Franck Chauvel, Stanislav Chren, Antonio Cicchetti, Federico Ciccozzi, Robert Clarisó, Tony Clark, Peter Clarke, Manuel Clavel, Rolland Colette, Nelly Condori-Fernández, Maxime Cordy, Carl Corea, Mario Cortes-Cornax, Jesús Sánchez Cuadrado, Alberto da Silva, Fabiano Dalpiaz, Marian Daun, Nancy Day, Alfonso de la Vega, Juan de Lara, Romulo De Oliveira, Julien DeAntoni, Patrick Delfmann, Romain Demangeon, Joerg Desel, Xavier Devroey, Juri Di Rocco, Davide Di Ruscio, Marcos Didonet Del Fabro, Aleksandar Dimovski, Crystal Din, Juergen Dingel, Zinovy Diskin, Lydie Du Bousquet, Francisco Duran, Alexander Egyed, Maged Elaasar, Eduard Enoiu, Romina Eramo, Huseyin Ergin, Maria Escalona, Lorenz Esch, Elisabet Estevez, Bedilia Estrada, Dirk Fahland, Michalis Famelis, Anna Rita Fasolino, Andreas Fellner, Peter Fettke, Barabara Fila, Hans-Georg Fill, John Fitzgerald, Ulrich Frank, Martin Fränzle, Agnès Front, Frederik Gailly, Antonio García-Domínguez, Gregory Gay, Sebastien Gerard, Marcus Gerhold, Mario Gleirscher, Martin Gogolla, Aniruddha Gokhale, Thomas Goldschmidt, Claudio Gomes, Cesar Gonzalez-Perez, Catarina Gralha, Peter Green, Joel Greenyer, Paul Grefen, Raffaela Groner, Georg Grossmann, Lars Grunske, Esther Guerra, Renata Guizzardi, Jens Gulden, Jin Guo, Simon Hacks, Sylvain Hallé, Stefan Hallerstede, Oystein Haugen, Jane Hayes, Xiao He, Regina Hebig, Reiko Heckel, Constance Heitmeyer, Loic Helouet, Martin Henkel, James Hill, Georg Hinkel, Nico Hochgeschwender, Jennifer Horkoff, Christopher Hoyle, Ludovico Iovino, Muhammad Zohaib Iqbal, Karim Jahed, Amin Jalali, Phillip James, Cyrille Jegourel, Manfred A. Jeusfeld, Christian Johansen, Monika Kaczmarek, Bernhard Kaiser, Sungwon Kang, Dimitris Karagiannis, Gabor Karsai, Evangelia Kavakli, Timo Kehrer, Udo Kelter, Wael Kessentini, Djamel Eddine Khelladi, Ferhat Khendek, Marite Kirikova, Stefan Klikovits, Alexander Knapp, Jan Kofron, Shekoufeh Kolahdouz-Rahimi, Dimitrios Kolovos, Dimitris Kolovos, Harald König, Nikolai Kosmatov, Stefan Kugele, Thomas Kühne, Akhil Kumar, Katsiaryna Labunets, Yngve Lamo, Kevin Lano, Xavier Le Pallec, Choonhwa Lee, Henrik Leopold, Shuai Li, Sotirios Liaskos, Crescencio Lima, Lukas Linsbauer, Guanjun Liu, Malte Lochau, Hugo A. López, Ernesto López-Mellado, Florian Lorber, Mass Soldal Lund, Fernando Macías, Frédéric Mallet, Beatriz Marín, Andrea Marrella, Florian Matthes, Raimundas Matulevicius, Claudio Menghi, Gergely Mezei, Faida Mhenni, Judith Michael, Miguel Mira da Silva, Raffaela Mirandola, Michael Möhring, Brice Morin, Sebastien Mosser, Christian Moya, Seyyedeh Atefeh Musavi, Gunter Mussbacher, Andreas Naderlinger, Pascal Négros, Bernd Neumayr, Phu Nguyen, Phuong Nguyen, Libero Nigro, Alexander Nolte, Arne Nordmann, Markus Nüttgens, Bentley Oakes, Peter Ölveczky, Andreas Opdahl, Mert Ozkaya, Richard Freeman Paige, Marc Pantel, Jovanka Pantovic, Nick Papoulias, Raul Pardo, Chris Partridge, Oscar Pastor, Patrizio Pelliccione, David Pereira, Diego Perez-Palacin, Barbara Pernici, Justyna Petke, Alfonso Pierantonio, Ingo Pill, Elias Pimenidis, Geert Poels, Pascal Poizat, Fiona Polack, Gregor Polančič, Luigi Pomante, Saheed Popoola, Sam Procter, Luise Pufahl, Ahsan Qamar, Truong Ho Quang, Ansgar Radermacher, Akshay Rajhans, Alexander Raschke, Muhammad Rashid, Daniel Ratiu, Gil Regev, Gianna Reggio, Manfred Reichert, Hajo Reijers, Wolfgang Reisig, Guizzardi Renata, Jan Oliver Ringert, Roberto Rodríguez-Echeverría, Marcela Ruiz, Adrian Rutle, Renaud Rwemalika, Aymen Saied, Gwen Salaun, Cesar Sanchez, Jesús Sánchez Cuadrado, Kurt Sandkuhl, Alceste Scalas, Axel Scheithauer, Johannes Schobel, Stefan Schönig, Ulrik Schultz, Christoph Schütz, Cristina Seceleanu, Ed Seidewitz, Lionel Seinturier, Bran Selic, Sagar Sen, Eltefaat Shokri, Natalia Sidorova, Anthony Simons, Monique Snoeck, Stefan Sobernig, Pnina Soffer, Hui Song, Wei Song, Matthew Stephan, Janis Stirna, Volker Stolz, Harald Störrle, Eleni Stroulia, Daniel Strüber, Patrick Stünkel, Arnon Sturm, Andreas Symeonidis, Eugene Syriani, Gabor Szarnyas, Jérémie Tatibouet, Michael Tautschnig, Ramin Tavakoli Kolagari, Martin Thibault, Matthias Tichy, Ulyana Tikhonova, Massimo Tisi, Saurabh Tiwari, Jake Tom, Hanh Nhi Tran, Javier Troya, Christos Tsigkanos, Valentín Valero, Antonio Vallecillo, Han van der Aa, Steven van Kervel, Simon Van Mierlo, Irene Vanderfeesten, Juan Manuel Vara, Tullio Vardanega, Daniel Varro, Gabriel Wainer, Pengyuan Wang, Barbara Weber, Ron Weber, Heike Wehrheim, Marco Wehrmeister, Jun Wei, Andrew Weinert, Georg Weissenbacher, Bernhard Westfechtel, Anton Wijs, Manuel Wimmer, Karsten Wolf, Uwe Wolter, Murray Woodside, Andreas Wortmann, Franz Wotawa, Jin-Long Wu, Sobhan Yassipour-Tehrani, Sira Yongchareon, Shin Yoo, Bahman Zamani, Anna Zamansky, Jelena Zdravkovic, Bernard P. Zeigler, Man Zhang, Alois Zoitl, Athanasios Zolotas, Steffen Zschaler, and Albert Zuendorf.

## Contents of this issue

The contents of this issue are as follows:

1. **Expert's Voice**
  “The triptych of conceptual modeling—A framework for a better understanding of conceptual modeling” by Heinrich C. Mayr and Bernhard Thalheim
2. **EMMSAD 2019 Special Section**
  Guest Editors: Iris Reinhartz-Berger and Jelena Zdravkovic
3. **Regular Papers**
  "Generation of hazard relation diagrams: Formalization and tool support" by Bastian Tenbergen and Thorsten Weyer
  "A framework for automated multi-stage and multi-step product configuration of cyber-physical systems" by Safdar Aqeel Safdar, Hong Lu, Tao Yue, Shaukat Ali, and Kunming Nie
  "On the benefits of file-level modularity for EMF models” by Karim Jahed, Mojtaba Bagherzadeh, and Jürgen Dingel

We wish you a Happy New Year with the hope that you enjoy reading the papers in this issue!

Huseyin Ergin, Jeff Gray, Bernhard Rumpe, and Martin Schindler

## Footnotes

1 The modeling community is indebted to the efforts of Eugene Syriani and Houari Sahraoui in hosting MODELS 2020 as a virtual conference.

